# Phylogenetic Community Structure and Niche Differentiation in Termites of the Tropical Dry Forests of Colombia

**DOI:** 10.3390/insects10040103

**Published:** 2019-04-10

**Authors:** Robin Casalla Daza, Judith Korb

**Affiliations:** 1 Departamento de Química y Biología, Universidad del Norte, Kilómetro 5 Antigua vía Puerto Colombia, 081007-Puerto Colombia, Colombia; 2 Evolutionary Biology & Ecology, University of Freiburg, Hauptstrasse 1, 79104-Freiburg, Germany; judith.korb@biologie.uni-freiburg.de

**Keywords:** community assembly, competition, isotopes, phylogenetic community, termites, trait mapping, tropical dry-forest, Colombia

## Abstract

The mechanisms that structure species communities are still debated. We addressed this question for termite assemblages from tropical dry forests in Colombia. These forests are endangered and poorly understood ecosystems and termites are important ecosystem engineers in the tropics. Using biodiversity and environmental data, combined with phylogenetic community analyses, trait mapping, and stable isotopes studies, we investigated the termite community composition of three protected dry forests in Colombia. Our data suggest that the structuring mechanisms differed between sites. Phylogenetic overdispersion of termite assemblages correlated with decreasing rainfall and elevation and increasing temperature. Food niche traits—classified as feeding groups and quantified by δ^15^N‰ and δ^13^C‰ isotope signatures—were phylogenetically conserved. Hence, the overdispersion pattern implies increasing interspecific competition with decreasing drier and warmer conditions, which is also supported by fewer species occurring at the driest site. Our results are in line with a hypothesis that decreased biomass production limits resource availability for termites, which leads to competition. Along with this comes a diet shift: termites from drier plots had higher δ^13^C signatures, reflecting higher δ^13^C values in the litter and more C4 plants. Our study shows how a phylogenetic community approach combined with trait analyses can contribute to gaining the first insights into mechanisms structuring whole termite assemblages.

## 1. Introduction

The drivers that structure communities are still debated [1,2,3,4]. Neutral and/or deterministic mechanisms have been proposed to explain community assembly [5,6]. Neutral models highlight that mainly stochastic processes drive local communities. Species are regarded as ecologically equivalent. At larger scales, meta-communities are influenced by dispersal, speciation, and extinction [7]. On the other side, deterministic models describe local communities as an “arranged” assembly of species, based on their physiology and their defined niches [8,9]. These are two extreme views of processes affecting community composition. Real communities will often fall along a continuum containing components of randomness, as well as determinism. Whether processes differ systematically between taxa, habitats, geographic regions, or biomes is still unclear. A major unsolved question that remains is whether tropical ecosystems differ systematically from, for example, temperate regions, and whether such differences in the structuring mechanisms can contribute to explaining their high species richness. However, such species-rich ecosystems are notoriously difficult to study due to their high number of undescribed species. Two approaches can contribute to overcoming this hurdle. First, molecular barcoding uses short genetic markers in an organism’s DNA to identify it as belonging to a particular species [10]. Hence, species can be identified more easily. Second, phylogenetic community analyses deduce potential mechanisms from the co-occurrences of species and their phylogenetic relatedness. They combine phylogenetic data with distributional and ecological data to assess whether and how communities of species differ from random assemblages with regard to evolutionary relatedness [1,2,3,4,5,6]. For instance, if species which locally co-exist are less closely related on the phylogeny than a random selection of species that could potentially co-occur (i.e., species from the regional species pool), this can indicate that interspecific competition can play an important role in structuring communities, given closely related species share the same niche traits [5,11]. As genetic sequence information and phylogenies are becoming increasingly available for many taxa, the use of phylogenetic community analyses can be a helpful and easy tool to gain first insights into potential assembly mechanisms. They also allow standardized comparisons between sites within a habitat, and between habitats, regions, or disturbed and natural areas, to inform about changes and similarities in community structuring mechanisms (for termites: [12,13]). Thus, both genetic barcoding and phylogenetic community analyses can help to gain first insights into community structuring mechanisms.

Tropical dry forests are the most threatened of all major tropical forest types [14,15]. Colombia has one of the best-conserved areas, mainly along the Caribbean coast [16]. These poorly studied ecosystems are threatened by land use, climate change [14], and urban expansion [15]. Termites (Termitoidea) are important ecosystem engineers of such tropical ecosystems [17,18]. They are important food sources for a wide range of species [19,20,21,22,23]. As the main macro-detritivores, they essentially contribute to the biotransformation of wood and litter into organic matter and the re-distribution of structural soil components [24]. Tropical forests produce plenty of dead plant material, which termites consume [25,26]. Four functional feeding groups are distinguished in termites [27,28]: dead wood-feeders (group I); dead wood, leaf, plant-litter feeders (group II); humus feeders (group III); and true soil feeders (group IV). No fungus-growing termites occur in the Neotropics, hence the differentiation of the feeding group IIF (i.e., fungus-feeder) is irrelevant [29]. Most termites of dry forests feed on twigs and litter and belong to feeding group I and II, while soil-feeders (sensu *Anoplotermes*-group) are relatively scarce in richness and abundance [26]. Nitrogen and carbon stable isotope ratios have been used in termites to elucidate feeding habits in more detail, including dietary preferences [30,31] and niche food differentiation [32,33,34,35]. However, most studies on stable isotopes have been conducted in savannas [31,33,35] and rainforests [32,36,37]. Information related to the trophic ecology of termites in Neotropical dry forests is unknown. We used isotope analyses to characterize the feeding niche of termites and combined this approach with phylogenetic community analyses to gain first insights into the mechanisms that may structure termite assemblages in dry tropical forests.

## 2. Materials and Methods

### 2.1. Study Sites and Termite Sampling

**Figure 1:**
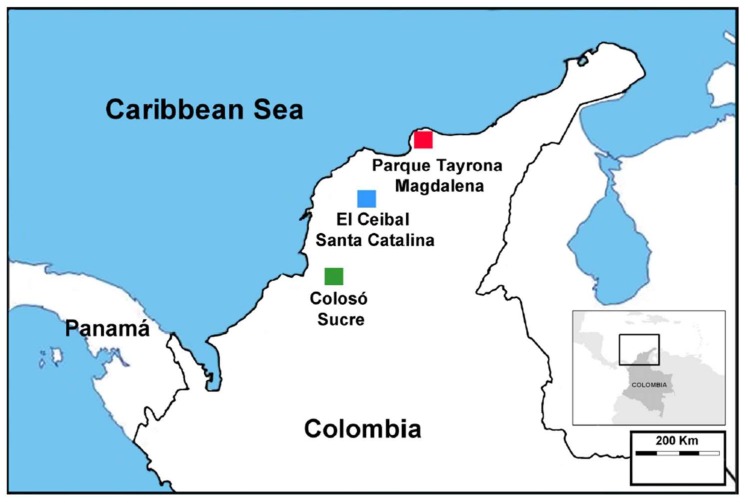
Study sites in Colombia: Reserva forestal de Coraza, Montes de María, Colosó, Sucre (green square); Parque Natural regional Bosque seco El Ceibal Mono Titi, Santa Catalina, Bolívar (blue square); and Parque Nacional Natural Tayrona, Santa Marta, Magdalena (red square).

Termites were studied in the Coraza Forestry Reserve ‘Colosó’ (hereafter, Colosó; Sucre; 9°31′51.3–9°32′24 N; 75°21′0–75°21′3.6 W ), the regional park ‘El Ceibal Mono Tití’ (hereafter, Ceibal; Santa Catalina, Bolívar; 10°37′40.8–10°38′13.2 N; 75°14′6–75°15′10.8 W), and the National Park ‘Tayrona’ (hereafter, Tayrona; Santa Marta, Magdalena; 11°19′19.2–11°18′43.2 N; 74°6′10.8–74°7′22.8 W) in Colombia (Figure 1). All these protected areas (hereafter ‘sites’) have important primary and secondary tropical dry forest [16,38,39,40,41,42].

In a former study, we characterized the termite communities of these three sites by determining species diversity and abundances and associating them with environmental variables [26]. We studied fives transect belts per site (hereafter called study plots) using the standardized belt transect sampling protocols of Jones and Eggleton [43] and Hausberger and Korb [44] developed for termites. We surveyed each site by sampling a transect measuring 2 m × 100 m, divided into twenty 2 m × 5 m sections. Each section was searched for termites on the ground, and in trees, mounds, and soil, (eight soil pits 15 cm ×15 cm ×15 cm depth) for 30 min by two trained persons. All study plots were randomly chosen and they were separated from each other by, on average, around 560 m (min: 225, max: 1043, SD +/− 253 m) in Colosó, 1074 m (min: 366, max: 1982, SD +/− 557 m) in Ceibal, and 1606 m (min: 508, max: 3157, SD +/− 985 m) in Tayrona. We also took soil and litter samples and retrieved climate data from WorldClim v 1.4 (http://www.worldclim.org/). The data layers were generated through the interpolation of average monthly climate data from weather stations on a 30 arc-second resolution grid (often referred to as a “1 km^2^” resolution). Variables included were monthly total precipitation and monthly mean temperature (for more details see http://www.worldclim.org). A combination of morphological and genetic analyses (molecular barcoding) revealed a total of 32 species for all three sites (Table A1).

### 2.2. Determination of Food Niche

To determine the feeding type and characterize the food niche using δ^13^C and δ^15^N isotope analyses, we used specimens and material from a former study [26]. δ^13^C and δ^15^N isotope analyses were done for termite workers, but also for soil and leaf litter (leaf and small pieces of wood), which is potential food for the termites. For each termite species, a whole termite was used. As in several other studies [30,31,34,45,46], we could not exclude the gut as this would have left too small amounts to conduct the analyses. Prior to analyses, termite samples were stored in ethanol (>99.5% Merck, Darmstadt, Germany). Three replicates (if available) per site were analyzed. Only workers were taken into account to eliminate the effect of inter-caste differences in isotopic values, which could bias cross-species comparisons [35,36]. In addition, workers are the caste that does the foraging and feeding within colonies. Five replicates of soil samples were collected from the top horizon (0–15 cm) at each site—one at each study plot—resulting in 15 samples in total for all study sites. Soil samples were cooled and directly dried after each field trip, and then sealed in plastic bags. Additionally, litter samples were collected on the ground; three samples were taken per study plot, including one at the start, in the middle, and at the end of each study plot, resulting in 15 replicates per site (45 in total). They included leaves, twigs, and dead wood. Like the soil samples, they were dried and kept cool, prior to analysis.

Soil samples were collected in each plot following the protocol by Pansu and Gautheyrou [47] and Osorio [48]. At a depth of 15 cm and a distance of 1 m parallel to each belt transect, three replicate soil samples were taken along the transect belt (one at the start, in the middle, and at the end of a belt transect), resulting in a total of 45 samples (3 sites × 5 belt transect × 3 replicates). Samples were prepared according to a protocol of the Centre for Stable Isotope Research and Analysis (KOSI) at the University of Göttingen (Germany). In short, all samples were dried at 60 °C for 24 h. Stones and gravel were removed before crushing samples and grounding them into fine powder. For soil and litter between 0.4 mg and 1.0 mg, one whole termite worker with gut was weighted, transferred into tin zinc capsules (HekaTech GmbH^®^, Wegberg, Germany), and sent to KOSI. 

Carbon and nitrogen stable isotope ratios were measured on an elemental analyzer (NA 1500, Fisons-Instruments, Rodano, Milan, Italy) and an isotope ratio mass spectrometer (Delta V Plus, Thermo Fisher Scientific, Bremen, Germany). Stable isotope ratios were expressed using the delta (δ) notation in ‰ according to:

$$\delta X=\left(\left(\frac{R\;sample}{R\;standard}\right)-1\right)\times 10^{3}$$

where *R sample* is the isotopic ratio of the sample (^13^C/^12^C or ^15^N/^14^N), *R standard* is the isotopic ratio of the international standard, and X is the respective element (^13^C or ^15^N). For ^13^C V-PDB and ^15^N, atmospheric nitrogen was used as the standard. Acetanilide (C_8_H_9_NO, Merck, Darmstadt, Germany) was used for internal calibration. For the amount of animal tissue analyzed per sample, precision of the measurement was about 0.1‰ for ^13^C and 0.2‰ for ^15^N. We calculated the mean and the standard deviation (SD) of all samples for each site.

### 2.3. Phylogenetic Community Analyses

**Figure A1:**
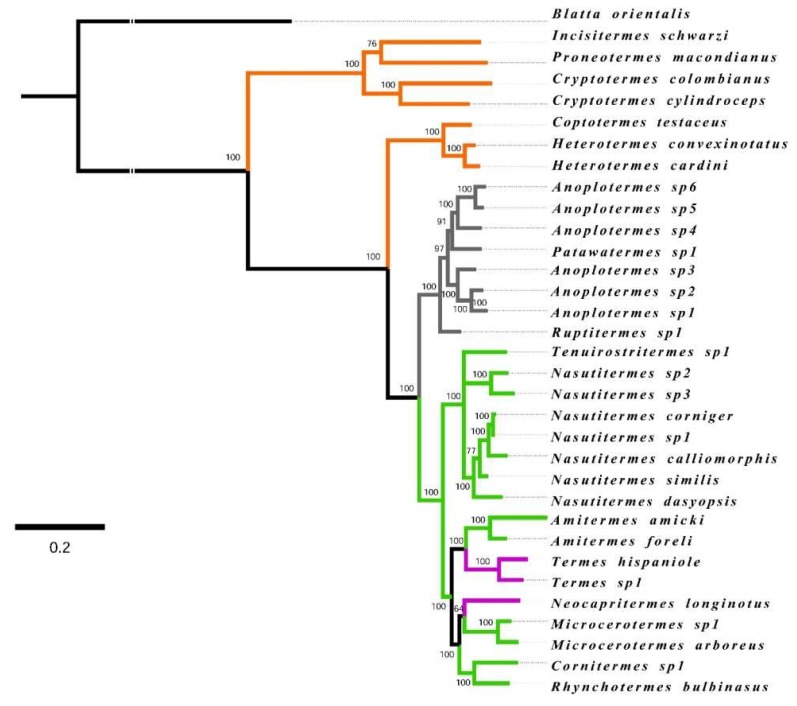
Phylogenetic tree inferred from COII, 12S, and 16S sequence data (nodes show Bayesian posterior probability support, BPP). Red: Kalotermitidae, Blue: Rhinotermitidae, Green: Termitidae. Outgroup: *Blatta orientalis*.

The species pool of the three study sites comprises 32 species that have been morphologically and genetically identified (Table A1: GenBank accession numbers MH09082–MH090914 and KU510330, KX267100, KX267099, KX267098, KX267095, KX267092) [26]. As the input tree for the phylogenetic community structure analyses, we used the combined COII, 12S, and 16S nucleotide sequences and performed a Bayesian approach using MrBayes 3.2.1. [49]. We pruned the tree prior to analysis to include only species of the regional species pool and only one representative per species in the tree (Figure A1, Table A2).

A commonly used index to quantify the phylogenetic structure of a local community is the Net Relatedness Index (NRI) [5]. It measures whether locally co-occurring species are phylogenetically more/less closely related than expected by chance. It uses phylogenetic branch length to measure the distance between each sample to every other terminal sample in the phylogenetic tree, hence the degree of overall clustering. It is calculated as the difference between the mean phylogenetic distance (MPD) of the tested local community (i.e., each study plots) and the MPD of the regional community (i.e., all 32 species identified for dry forests in this region), divided by the standard deviation of the latter. NRI values close to zero indicate random community assembly, which may imply that neutral processes are important in structuring communities. Large positive values reflect phylogenetic clustering of co-occurring species (i.e., co-occurring species are more related than expected by chance), whereas low negative values point to over-dispersion (i.e., co-occurring species are less related than expected by chance) [1].

Depending on whether niche-relevant traits, such as the feeding niche, are evolutionary labile or conserved, the NRI values can hind at different assembly processes [3]: For instance, conserved traits and over-dispersion can indicate that interspecific competition plays an important role in structuring communities. We analyzed the phylogenetic community structure with PHYLOCOM 4.2 [5]. As the input tree, we used the Bayesian inference tree in combination with abundance data for all species. We conducted two analyses, including one testing the local assemblages against the regional species pool (all species found during this study) and one site specific analysis in which we tested the local assemblages against the species occurring at a specific study site. We tested whether our data significantly deviated from null models using the independent swap algorithm on occurrence data [50]. This algorithm creates swapped versions of the sample/species matrix while constraining row (species) and column (occurrence) totals to match the original matrix. We used two-tailed significant rank tests as suggested by Webb et al. [5] to determine if observed values differed significantly from the null model (e.g., with 9999 randomizations, rank values equal or higher than 9750 or equal or lower than 250 are statistically significant at *p* = 0.05). 

### 2.4. Mapping Food Niche Traits on Phylogeny

In order to interpret the results of the phylogenetic community analysis, it is necessary to know whether the studied food niche traits were phylogenetically conserved or labile. To determine this, we conducted two analyses, including one with feeding groups and one for isotope signatures. We used (i) the feeding groups and (ii) the mean of the δ^13^C and δ^15^N values calculated over all collection sites for each species as character states and the phylogenetic tree from Casalla & Korb [26] (which was inferred from molecular sequence data) as the input, and performed ancestral state reconstruction (ASR). For inferring ancestral states, we used Mesquite version 3.04 [51,52], in particular, the module ‘Parsimonious Ancestral States: ‘Parsimony unordered’ for the categorical feeding group data and ‘Parsimony Squared’ for quantitative isotope data.

## 3. Results

### 3.1. Phylogenetic Community Structure

**Figure 2:**
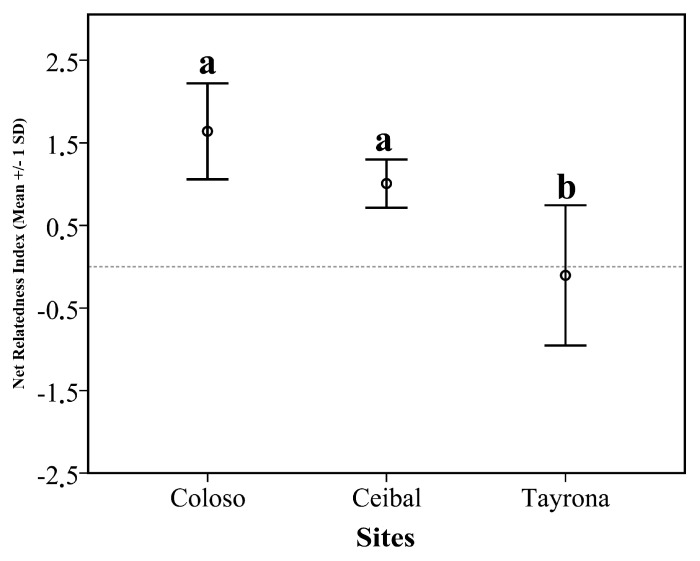
NRI (Net Relatedness Index) of study sites in Colombia. High positive values indicate phylogenetic clustering, while negative values indicate phylogenetic overdispersion. Shown are bars with mean (+/− 1 SD). Different letters indicate significant differences (ANOVA: F_2, 12_ = 15.75; and *p* = 0.011, *p* < 0.001 respectively, Tukey *p* < 0.001, Table A3a).

**Figure 3:**
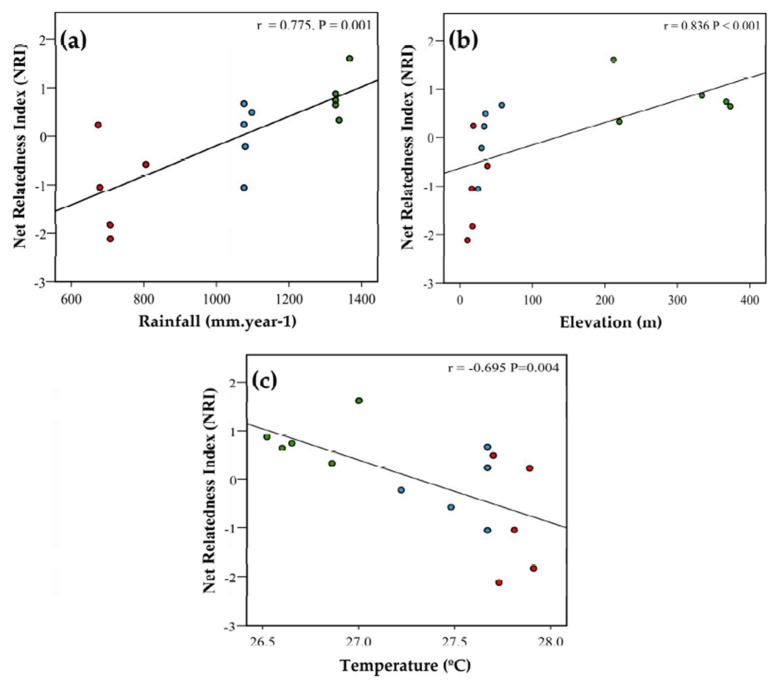
Pearson correlations between Net Related Index of study plots and (**a**) rainfall (mm year^−1^), r = 0.862, *p* < 0.001; (**b**) elevation r = 0.626, *p* < 0.012; and (**c**) temperature (°C) r = −0.648, *p* = 0.009.

Overall, the NRI values which measured the phylogenetic structure of the termite assemblages across the regionals species pool ranged from −0.82 to 2.45 (Table A3a). NRI values did not differ significantly from random expectation, except for one site in Colosó (Colosó 5), which showed significant signs of phylogenetic clustering (Table A3a). Both sites, Colosó and Ceibal, had significantly higher NRI values than Tayrona, where species were more phylogenetically overdispersed (Figure 2, Table A3a). At the study plot level, NRI values did not correlate with species richness (Pearson correlation r = 0.339, *p* = 0.217). However, NRI values significantly increased with rainfall at a study plot (r = 0.862, *p* < 0.001; Figure 3a) and its elevation (r = 0.626, *p* = 0.012; Figure 3b). Contrarily, NRI decreased with temperature (r = −0.648, *p* = 0.009; Figure 3c).

When analyzing the NRI values generated by using the site-specific termite pool, we did not detect significant effects of the abiotic variables (Table A3b).

A mixed effect model of NRI and the three abiotic variables (rainfall, elevation, and temperature), using site as the random factor, showed significant effects for temperature only (Table 1). Mixed effects using fine-scale were not significant (*p* > 0.082, Table A3b).

### 3.2. Isotopes Stable Analyses

**Figure 4:**
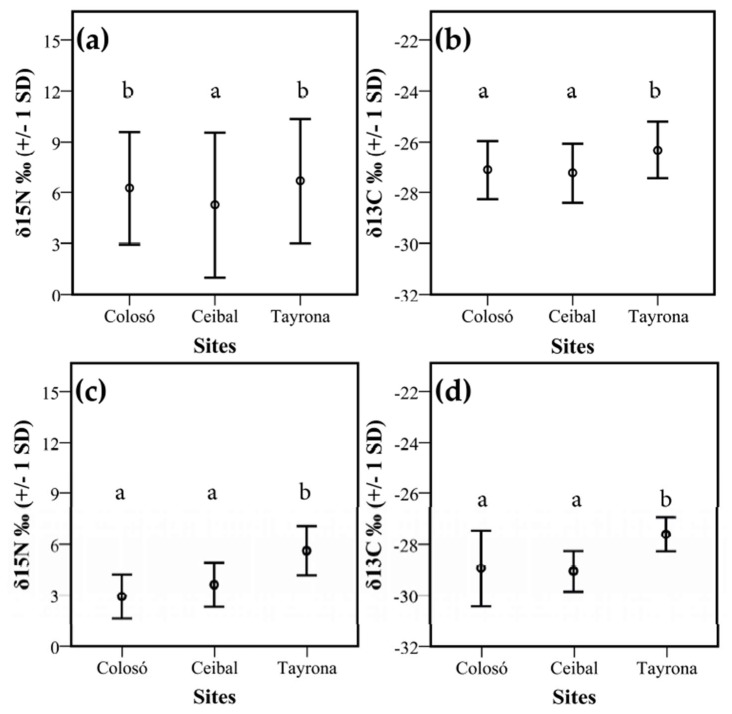
Isotope signatures of δ^15^N and δ^13^C between study sites. Shown are mean values +/− 1 SD for termite (**a**) δ^15^N‰ and (**b**) δ^13^C‰ and litter (**c**) δ^15^N‰ and (**d**) δ^13^C‰ over all three sites: Colosó, Ceibal, and Tayrona. Different letters indicate significant differences between sites (Tukey test, *p* < 0.05).

**Figure A2:**
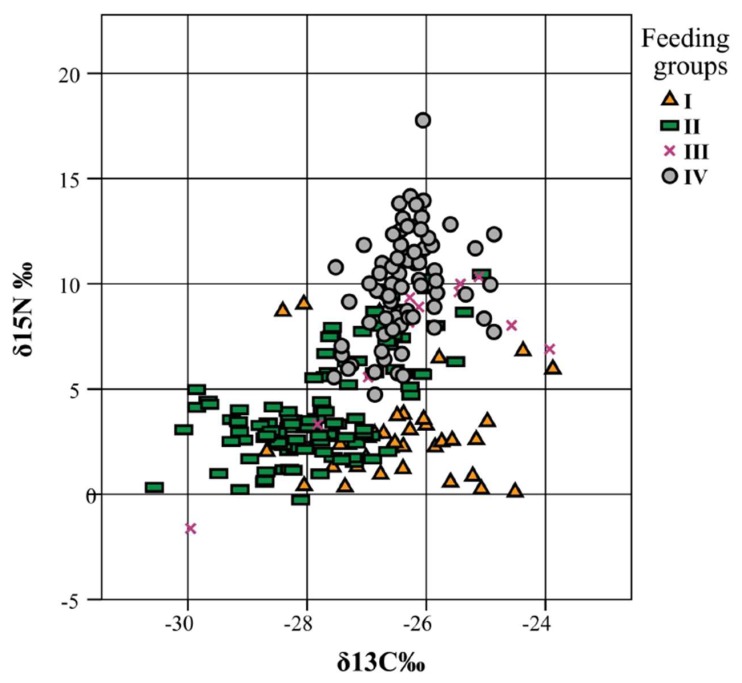
Scatterplot between δ^15^N and δ^13^C. Symbols: Orange square: feeding group I; green circle: feeding group II; purple circle: feeding group III; black circle: feeding group IV.

**Figure A3:**
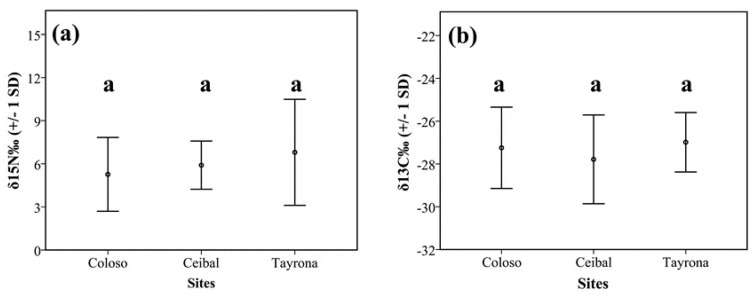
Boxplot showing mean values for (**a**) δ^15^N‰ and (**b**) δ^13^C‰ +/− 1 SD for soil between sites. Soil samples did not differ significantly between sites for δ^15^N‰ (ANOVA: F_2, 12_ = 1.55; *p* = 0.253) and δ^13^C‰ (ANOVA: F_2, 12_ = 1.01; *p* = 0.391).

δ^15^N values of termites were highly variable, ranging from −1.6‰ to 17.8‰ (mean 6.0 ‰ +/− 1 SD 3.8, Figure A2, Table A4). Both Colosó and Tayrona differed significantly from Ceibal (F_2, 12_ = 4.78; *p* = 0.03, Figure 4a, Table A5). Additionally, the δ^13^C values of termites were highly variable (mean −27.0‰ +/− 1 SD 1.2; Min: −30.6, Max: −23.9). The termites from both Colosó (mean −27.1‰ +/− 1 SD 1.1) and Ceibal (mean −27.2‰ +/− 1 SD 1.2) had significantly lower δ^13^C values than those from Tayrona (mean −26.3 ‰ +/− 1 SD 1.1) (F_2, 12_ = 5.32; *p* = 0.022, Figure 4b, Table A6). In addition, the δ^15^N and δ^13^C signatures for litter samples were also significantly lower at these two sites than at the dry site Tayrona (ANOVA: F_2, 72_ = 26.90; *p* < 0.001, Tukey test *p* < 0.001, Figure 4c,d). Soil samples did not differ significantly between sites for δ^15^N (ANOVA: F_2, 12_ = 1.55; *p* = 0.253) and δ^13^C (ANOVA: F_2, 12_ = 1.01; *p* = 0.391). (Figure A3).

**Figure 5:**
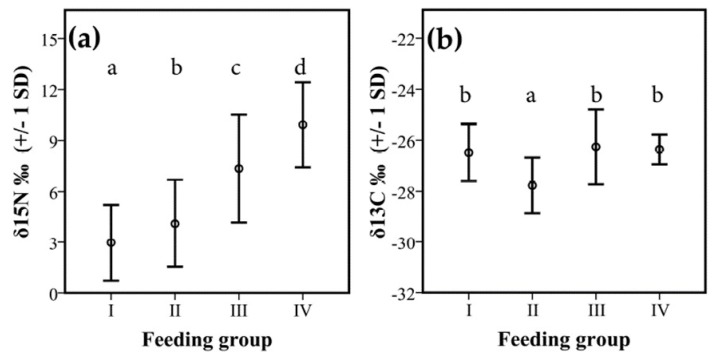
Difference in isotope signatures between feeding groups. Shown are mean values +/− 1 SD for (**a**) δ^15^N‰ and (**b**) δ^13^C‰ between the four feeding groups: I: dead wood-feeders; II: dead wood, leaf, plant-litter feeders; III: humus feeders; and IV: true soil feeders. Different letters indicate significant differences in litter samples (*p* < 0.05, Table A3 and Table A4).

There were also significant differences in isotope signatures between feeding groups. δ^15^N values differed significantly between all groups (F_1, 212_ = 23.80, *p* < 0.001) (Figure 5a). Species from feeding group I had significantly lower δ^15^N values than those of feeding group II (*p* = 0.047), III (*p* < 0.001), and IV (*p* < 0.001). Termites from feeding group II had significantly lower δ^15^N values than those of the other feeding groups, III (*p* < 0.001) and IV (*p* < 0.001). Termites from feeding group III had significantly lower from feeding group IV (*p* < 0.001). Thus, there was a gradual increase of δ^15^N over the feeding groups.

For the δ^13^C, there were less strong differences between groups. Only feeding group II had significantly lower values than all other feeding groups (mean: −28.2‰ +/− 1 SD 1.0; F_1, 212_ = 9.34, *p* = 0.003, Figure 5b).

**Figure 6:**
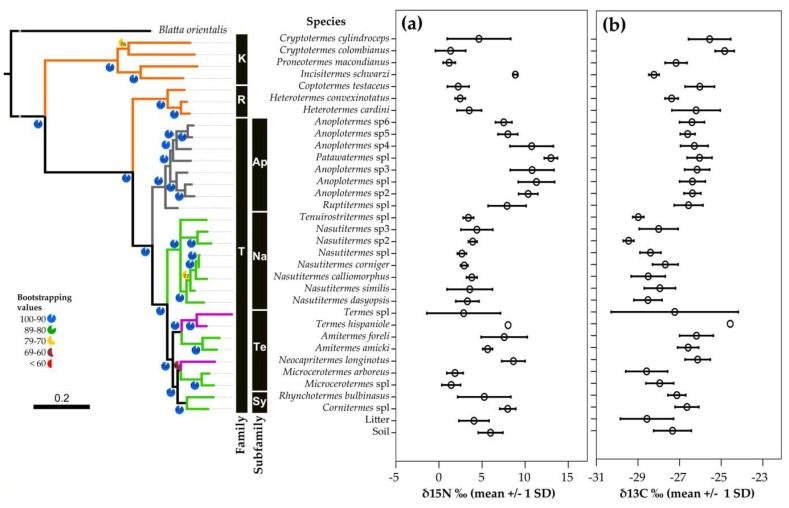
Phylogenetic relationships between the studied termites and their associated δ^15^N and δ^13^C signatures, together with that of litter and soil. Bootstrap values close to nodes. (**a**) Shown are bars with mean for δ^15^N‰ +/− 1 SD over all three study sites. (**b**) Bars with mean for δ^13^C‰ +/− 1 SD over all three study sites. Orange: Feeding group I, Green: Feeding Group II, Purple: Feeding group III, Grey: Feeding group IV. Families: K: Kalotermitidae, R: Rhinotermitidae, and T: Termitidae. Subfamilies: Ap: Apicotermitinae, Na: Nasutitermitinae, Te: Termitinae, and Sy: Syntermitinae.

**Figure A4:**
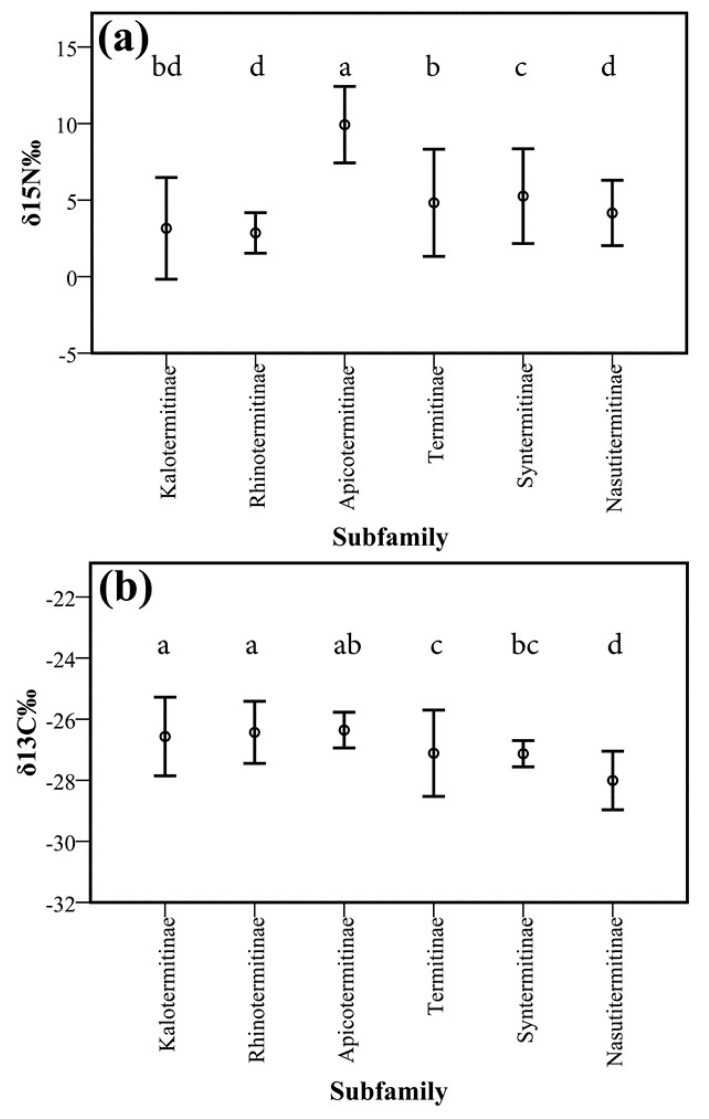
Boxplot showing mean +/− 1 SD for δ^15^N‰ (**a**) and δ^13^C‰ (**b**) by termite subfamily. Different letters indicate significant differences (*p* < 0.05).

There were also differences in δ^15^N and δ^13^C signatures between taxonomic groups (Figure 6 and Figure A4, Table A4, Table A5 and Table A6). The lower termite species from the Rhinotermitidae (2.8‰ +/− 1 SD 1.3) and Kalotermitidae (3.2‰ +/− 1 SD 3.3) had the lowest δ^15^N values, which were significantly lower for the Rhinotermitidae than for species from the Termitidae (6.6‰ +/− 1 SD 3.8) (F_3_, _212_ = 14.49; *p* < 0.001). Within the Termitidae, the δ^15^N also reflected the different feeding groups. Wood feeders of feeding group II, such as *Microcerotermes*, generally had lower values than humus and soil feeders, with the highest δ^15^N values occurring in the *Anoplotermes*-group (Figure 6). Some species (e.g., *Cryptotermes cylindroceps*, *Rhynchotermes bulbinasus*, and *Termes* sp1) had very high intraspecific variability (Figure 6a). 

For δ^13^C, the subterranean Rhinotermitidae had the highest values (−26.4‰ +/− 1 SD 1.0), which were significantly higher than for species from the Termitidae (−27.1‰ +/− 1 SD 1.2, Figure 6b). 

### 3.3. Mapping Food Niche Traits on Phylogeny

**Figure 7:**
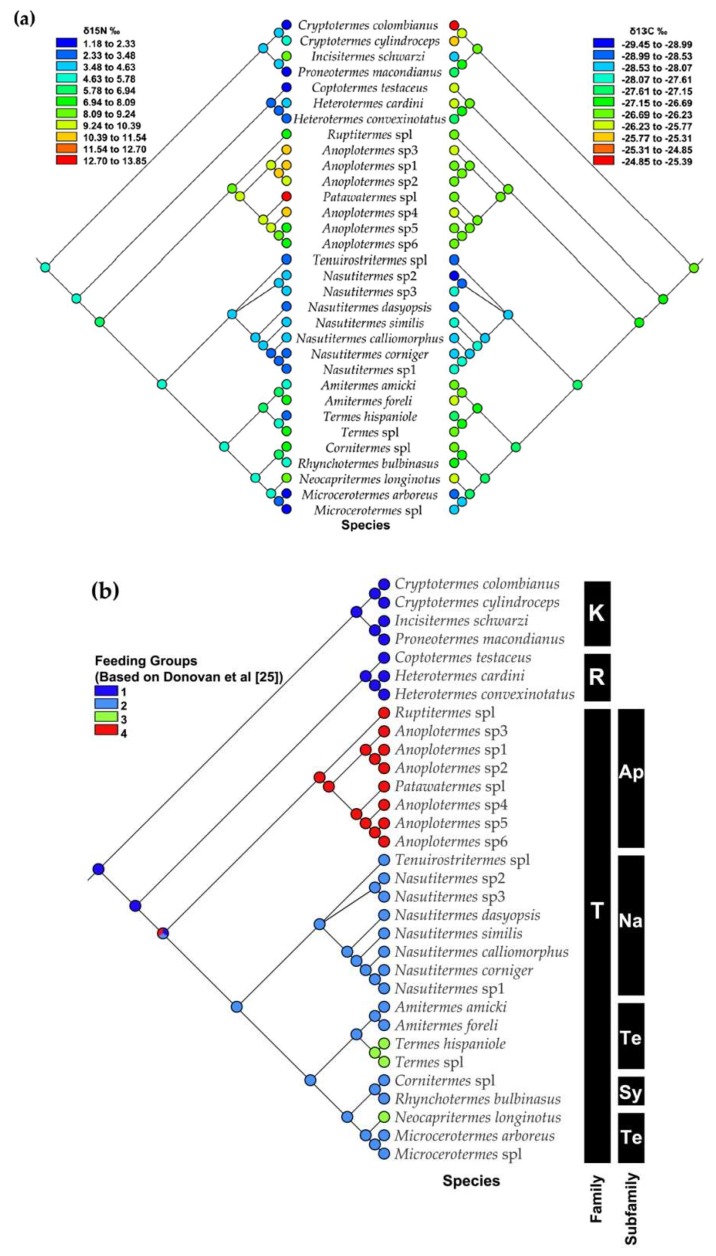
(**a**) Mirrored phylogenetic trees for a subset of 32 termites species, showing parsimony reconstruction of quantitative δ^15^N values from tropical dry forest termites (left-side tree) compared to quantitative δ^13^C (right-side tree), (**b**) ancestral states for categorical feeding groups based on Donovan et al., 2001 [25]; Dark blue represents feeding group I, light blue represents feeding group II (down-side tree), green represents feeding group III, and red represents feeding group IV. Ancestral states represented by colors at the nodes of the phylogeny observed at the tips are circled at each node. Families: K: Kalotermitidae, R: Rhinotermitidae, and T: Termitidae. Subfamilies: Ap: Apicotermitinae, Na: Nasutitermitinae, Te: Termitinae, and Sy: Syntermitinae.

Our analyses showed that food niches, measured as feeding group membership and δ^15^N and δ^13^C signatures, are phylogenetically conserved traits in the studied species. Closely related species share the same feeding group (right part of Figure 7a,b). Among the studied termites, group IV soil feeders evolved only once from group II plant litter feeders or group I wood feeders. Interestingly, group IV soil feeders do not seem to have evolved from group III soil feeders (and vice versa). At the fine-scale of the δ^15^N and δ^13^C signatures, the δ^15^N signal reflects the feeding group pattern well, except for a few species, such as *Incisitermes schwarzi* and *Termes* sp.1 (Figure 7a, left part). Thus, the δ^15^N signature has a strong phylogenetic signal, with closely related species sharing similar signatures (Figure 7a). The δ^13^C signatures are also phylogenetically conserved, but their pattern does not reflect that of the feeding groups (Figure 7b, right part). The Kalotermitidae (feeding group I) (especially *Cryptotermes*) had the highest δ^13^C values, while Nasutitermitinae and *Microcerotermes* (both feeding group II, but independent transitions) had the lowest values.

## 4. Discussion

Our results imply that mechanisms which structure the termite assemblages differ between sites, with interspecific competition being more important at drier and warmer, lower-altitude plots (Figure 2 and Figure 3). This is in line with a hypothesis of food-limitation becoming important in such areas. 

### 4.1. Mechanisms Structuring Termite Assemblages

Inferred from the phylogenetic community analyses, the assembly processes in the studied Colombian dry forests seem to differ between sites. The driest and lowest elevation site, Tayrona, had termite assemblages that were phylogenetically more overdispersed than those of the other sites (Figure 2). Overall, phylogenetic overdispersion correlated negatively with rainfall and elevation of study plots and positively with temperature (or vice versa, phylogenetic clustering increased with rainfall and elevation, but decreased with temperature) (Figure 3). The mixed model analyses including all three environmental analyses together, revealed that rainfall had the strongest and only significant effect (Table 1). That the results are non-significant when using the NRI values generated with the site-specific species pools as the reference supports the conclusion that the pattern is mainly driven by abiotic differences between study sites and not by differences between study plots within a site. To interpret this pattern ecologically requires knowledge of whether niche traits are phylogenetically conserved or labile. For conserved traits (i.e., closely related species share traits), overdispersion implies that species which share the same niche traits are less likely to co-exist than species that differ in these traits. Our phylogenetic trait mapping analyses showed that the food niche traits are phylogenetically conserved for the studied termite assemblages. This indicates that interspecific competition is more important in Tayrona, and in general, at the drier and warmer low-elevation study plots, than at the more humid and slightly colder high-elevation plots. In line with this, there are fewer termite species at the Tayrona plots [26]. In tropical dry forests, rainfall rather than temperature limits vegetation growth [53], with the latter still being optimal for plant growth at all sites (mean 26.5–27.7 °C). In line, the mixed model analyses only showed a significant effect for rainfall. Thus, there is lusher vegetation with higher biomass production in Colosó [54] and Ceibal [55,56] than in Tayrona [41], where Euphorbiaceae and Cactaceae are common. This supports the hypothesis that food is a limiting resource over which termites compete at Tayrona. Furthermore, other studies found evidence that food can be a limiting resource (i.e., dead plant material) for termites [57], which can lead to intra- and interspecific competition [57,58,59,60,61]. In a West African savannah, annual fires reduce the availability of dead plant material, so that the addition of dried grass after the fires leads to an increase in the number of sexual produced by the dominant mound building termite Macrotermes bellicosus [57].

This does not seem to be the case at the two other sites. NRI values close to zero imply that assemblages at Colosó and Ceibal do not differ much from random associations. The fact that the NRI values at the plot scale did not differ significantly from random expectation might be due to the low numbers of species per plot. Note, that the species number is the sample size for these tests and that small sample sizes are associated with low statistical power, hence making it unlikely to detect significant effects. One plot in Colosó even showed signs of phylogenetic clustering, implying that environmental variables select for a certain subset of termites at this specific plot, which was characterized by huge trees with a dense canopy and high humidity. Four *Nasutitermes* species and four species of the *Anoplotermes*-group co-existed in this plot. Several studies from Neotropical rain forests have also shown that these closely related species commonly co-occur [62,63,64].

How wide-spread are the implicated structuring mechanisms in termites? Comparable studies are rare. Most research on termite communities has concentrated on describing local or regional assemblages and testing associations between termite diversity and variables such as fire, disturbance, or elevation gradients [65,66,67,68]. The few studies that have addressed community processes in more detail in termites have often concentrated on a subset of species. They found evidence for interspecific competition in structuring assemblies at the local scale [32,44,58,69,70]. The only studies directly comparable to our study come from Africa. There phylogenetic community analyses imply random processes [49], but also evidence for interspecific competition [12,71] and environmental filtering [13], depending on the study site, disturbance regime, and presence of a dominant mound building termite species. Thus, we currently cannot derive any general conclusions and more similar studies are needed.

### 4.2. Food Niche: Isotopes and Termite Feeding Groups

In our study, also the isotope analyses, which admittedly included the termites’ gut, of the litter support the notion of vegetation differences in Tayrona as the δ^15^N and δ^13^C values were significantly higher at this site. Interestingly, the soil signatures did not differ between sites. For δ^13^C, but not δ^15^N, the shift in the litter signatures between sites is reflected in the termites’ isotope signal (Figure 5). Differences in δ^13^C mainly reflect varying proportions of C_3_ and C_4_ plants in the food of termites, while differences in δ^15^N indicate the diverse proportions of variably humified food resources [32,34,36]. The higher δ^13^C values at Tayrona reflect the presence of more grasses and especially abundant Euphorbiaceae [41], which are all C_4_ plants. In addition, due to the proximity of the sea and the associated salinity- and water-stress, also C_3_ plants have higher δ^13^C values [72,73]. 

In general, our isotope signatures were similar to those found for termites in other forests [31,32]. However, a study in an African savanna with many fungus-growing termites revealed higher δ^13^C values at the upper range and a lower δ^15^N signature at the lower range, reflecting a higher proportion of C4 grasses in the habitat and a broader food niche spectrum of fungus-growing termites [33,37]. 

Reflecting a humification gradient, the four commonly recognized feeding groups identified by Donovan et al. [27] can generally be distinguished by isotope signatures, especially δ^15^N [34,37]. Nevertheless, there can be limitations, as the rain forest and savanna revealed, which did not recover a discontinuity in δ^15^N values between group I and II or between III humus- and group IV soil-feeders [74]. Our current study separated all feeding groups for δ^15^N signatures (Figure 5a, Figure 6a and Figure 7a), supporting Donovan´s feeding group concept (Figure 7b). However, for δ^13^C, no such gradient was revealed and only feeding type II had δ^13^C values that were lower and differed significantly from all other feeding types (Figure 5b, Figure 6b and Figure 7b). This implies that isotope studies are required to reliably determine the food niches of termites. 

## 5. Conclusions

Mechanisms that structure termite assemblages in dry forests are complex. Both neutral and deterministic processes seem to be present, with decreasing rainfall probably leading to interspecific competition and a reduction of species caused by limited food availability. More studies are needed that specifically test for these mechanisms. However, our study shows how a phylogenetic community approach combined with trait analyses can contribute to gaining the first insights into mechanisms structuring whole termite assemblages. 

## Figures and Tables

**Table 1:** Estimates of the mixed effect model of NRI and the three abiotic variables rainfall, elevation, and temperature. The random variable “Sites” was not considered within the model, and variability was insignificant (standard deviation = 2.30e^−05^).

Variable	Estimate	Standard Error	df	t-Value	*p*-Value
Intercept	0.787	0.142	9	5.55	<0.001
Rainfall	0.885	0.290	9	3.05	0.014
Temperature	0.395	0.437	9	0.90	0.391
Elevation	0.263	0.352	9	0.74	0.475

## Appendix A

**Table A1:** GenBank accession numbers for all species for the COII, 12S, and 16S sequences.

Species	GenBank		
	Accession COII	Accession 12S	Accession 16S
*Blatta orientalis*	DQ874267.1	DQ87403.1	U17774.1
*Amitermes amicki*	MH090825	MH090861	MH090885
*Amitermes foreli*	MH090826	MH090860	MH090886
*Anoplotermes* sp1	MH090827	MH090876	MH090887
*Anoplotermes* sp2	MH090828	MH090881	MH090888
*Anoplotermes* sp3	MH090829	MH090878	MH090889
*Anoplotermes* sp4	MH090831	MH090880	MH090891
*Anoplotermes* sp5	MH090832	MH090877	MH090892
*Anoplotermes* sp6	MH090833	MH090879	MH090893
*Coptotermes testaceus*	MH090834	MH090857	MH090894
*Cornitermes* sp1	MH090835	MH090866	MH090895
*Cryptotermes colombianus*	KU510330	KX267100	KX267099
*Cryptotermes cylindroceps*	MH090836	MH090856	MH090896
*Heterotermes cardini*	MH090837	MH090859	MH090897
*Heterotermes convexinotatus*	MH090838	MH090858	MH090898
*Incisitermes schwarzi*	MH090839	MH090855	MH090899
*Microcerotermes arboreus*	MH090840	MH090872	MH090900
*Microcerotermes* sp1	MH090841	MH090871	MH090901
*Nasutitermes corniger*	MH090846	MH090882	MH090906
*Nasutitermes dasyopsis*	MH090843	MH090869	MH090903
*Nasutitermes similis*	MH090844	MH090873	MH090904
*Nasutitermes callimorphus*	MH090845	MH090870	MH090905
*Nasutitermes* sp1	MH090842	MH090868	MH090902
*Nasutitermes* sp2	MH090848	MH090862	MH090908
*Nasutitermes* sp3	MH090849	MH090863	MH090909
*Neocapritermes longinotus*	MH090847	MH090867	MH090907
*Patawatermes* sp1	MH090830	MH090874	MH090890
*Proneotermes macondianus*	KX267098	KX267095	KX267092
*Rhynchotermes bulbinasus*	MH090850	MH090865	MH090910
*Ruptitermes* sp1	MH090851	MH090875	MH090911
*Tenuirostritermes* sp1	MH090852	MH090864	MH090912
*Termes hispaniolae*	MH090853	MH090883	MH090913
*Termes* sp1	MH090854	MH090884	MH090914

**Table A2:** Occurrences of termites per study plot (100 m).

Species	F	SF	FG	CO1	CO2	CO3	CO4	CO5	CE1	CE2	CE3	CE4	CE5	TA1	TA2	TA3	TA4	TA5	TOT
*Cryptotermes colombianus*	K		I	0	0	0	0	0	0	0	0	0	0	1	0	0	0	0	1
*Cryptotermes cylindroceps*	K		I	0	0	0	0	0	0	0	0	0	0	2	0	2	0	3	7
*Incisitermes schwarzi*	K		I	0	0	0	0	0	0	0	0	0	2	0	0	0	0	0	2
*Proneotermes macondianus*	K		I	0	0	0	1	0	0	0	7	0	0	0	0	0	1	0	9
*Coptotermes testaceus*	R		I	1	0	0	5	0	4	0	0	6	0	0	0	0	0	0	16
*Heterotermes cardini*	R		I	5	6	6	1	7	16	12	5	0	3	20	2	8	4	11	106
*Heterotermes convexinotatus*	R		I	0	0	0	0	0	0	0	0	8	0	0	0	0	0	0	8
*Anoplotermes* sp1	T	Ap	IV	0	0	0	0	2	0	0	9	2	10	0	0	0	0	0	23
*Anoplotermes* sp2	T	Ap	IV	2	5	7	6	1	0	0	0	0	0	0	0	0	0	0	21
*Anoplotermes* sp3	T	Ap	IV	1	0	0	7	19	0	0	3	0	12	1	1	1	0	0	45
*Anoplotermes* sp4	T	Ap	IV	0	3	2	2	0	0	0	1	0	4	1	0	3	1	1	18
*Anoplotermes* sp5	T	Ap	IV	0	0	0	0	3	1	1	0	0	1	0	0	0	0	0	6
*Anoplotermes* sp6	T	Ap	IV	2	15	1	8	3	0	0	0	0	0	0	0	0	0	0	29
*Patawatermes* sp1	T	Ap	IV	0	1	0	0	0	0	0	0	0	4	0	0	1	0	0	6
*Ruptitermes* sp1	T	Ap	IV	0	0	1	0	2	1	1	0	0	0	6	5	8	1	4	29
*Nasutitermes corniger*	T	Na	II	16	13	0	3	4	0	0	0	0	0	0	0	0	0	0	36
*Nasutitermes dasyopsis*	T	Na	II	0	0	0	0	1	1	1	2	1	5	21	1	5	0	9	47
*Nasutitermes similis*	T	Na	II	6	0	0	10	1	0	0	0	0	0	0	0	0	0	0	17
*Nasutitermes callimorphus*	T	Na	II	0	0	0	0	0	0	0	18	0	11	0	0	0	0	0	29
*Nasutitermes* sp1	T	Na	II	0	0	28	18	35	0	0	0	0	0	0	0	0	0	0	81
*Nasutitermes* sp2	T	Na	II	0	0	0	2	0	0	0	0	0	0	0	0	0	0	0	2
*Nasutitermes* sp3	T	Na	II	0	0	0	1	0	0	1	0	0	0	4	0	0	2	0	8
*Tenuisrostritermes* sp1	T	Na	II	0	0	0	3	0	0	0	0	0	0	0	0	0	0	0	3
*Amitermes amicki*	T	Te	II	0	0	0	0	0	0	0	0	0	0	1	1	2	1	0	5
*Amitermes foreli*	T	Te	II	0	0	1	3	0	6	5	0	8	24	7	0	3	1	1	59
*Microcerotermes arboreus*	T	Te	II	0	0	0	0	0	15	0	19	29	46	0	0	0	0	0	109
*Microcerotermes* sp1	T	Te	II	8	12	1	16	22	6	37	6	47	21	35	24	17	11	22	285
*Neocapritermes longinotus*	T	Te	III	3	1	2	6	1	3	5	0	0	0	0	0	3	0	0	24
*Termes hispaniolae*	T	Te	III	3	0	0	0	0	0	0	0	0	0	0	0	0	0	0	3
*Termes* sp1	T	Te	III	0	0	0	0	0	0	0	0	0	0	0	0	2	0	0	2
*Cornitermes* sp1	T	Sy	II	7	21	16	6	3	0	0	0	0	0	0	0	0	0	0	53
*Rhynchotermes bulbinasus*	T	Sy	II	0	0	0	0	0	0	11	0	0	3	0	0	0	0	0	14
Number of species	32			11	9	10	17	14	9	9	9	7	13	11	6	12	8	7	
Number of occurrences				54	77	65	98	104	53	74	70	101	146	99	34	55	22	51	1103

(F) = Family: (K) = Kalotermitidae; (R) = Rhinotermitidae; (T) = Termitidae. (SF) = Subfamily: (Ap) = Apicotermitidae; (Na) = Nasutitermitinae; (Te) =Termitinae; (Sy) = Syntermitidae. (FG) = Feeding groups follow Donovan et al. 2001: (I) = wood; (II) = leaf-litter; (III) = humus; (IV) = soil feeders.

Table A3 (**a**) Results of phylogenic local community analyses from all the study plots using the regional species pool of all species occurring in the study as a reference for the null models. None of the NRI values except for Coloso_5 differed significantly from a random assemblage. (**b**) Results of the phylogenic community analyses using the site-specific termite pools as references. None of the NRI values differed significantly from a random assemblage.

**Table:** (**a**)

Plot	Taxa	NRI
Coloso_1	11	1.456
Coloso_2	9	1.411
Coloso_3	10	1.594
Coloso_4	17	1.282
Coloso_5	14	2.452
Ceibal_1	9	1.18
Ceibal_2	9	1.313
Ceibal_3	9	0.009
Ceibal_4	7	0.886
Ceibal_5	13	0.738
Tayrona_1	11	−0.821
Tayrona_2	6	0.798
Tayrona_3	12	0.412
Tayrona_4	7	−0.576
Tayrona_5	7	−0.340

**Table:** ANOVA from all the study plots at the regional level (*p* < 0.001).

NRI	Sum of Squares	df	Mean Square	F	*p*-Value
Between sites	7.80	2	3.900	15.76	<0.001
Within sites	2.97	12	0.248		
Total	10.77	14

**Table:** Multiple comparisons from all the study plots at the regional level.

(I) Site	(J) Site	Mean Difference (I–J)	Std. Error	*p*-Value
Colosó	Ceibal	0.63	0.31	0.152
	Tayrona	1.74	0.31	<0.001
Ceibal	Tayrona	1.11	0.31	0.011

**Table:** (**b**)

Plot	Taxa	NRI
Coloso_1	11	0.408
Coloso_2	9	0.549
Coloso_3	10	0.71
Coloso_4	17	−0.856
Coloso_5	14	1.446
Ceibal_1	9	1.023
Ceibal_2	9	1.382
Ceibal_3	9	−0.219
Ceibal_4	7	0.834
Ceibal_5	13	0.659
Tayrona_1	11	−0.818
Tayrona_2	6	0.828
Tayrona_3	12	0.449
Tayrona_4	7	−0.522
Tayrona_5	7	−0.336

**Table:** ANOVA (finer scale). None of the NRI values differed significantly between sites.

NRI	Sum of Squares	df	Mean Square	F	*p*-Value
Between sites	1.714	2	0.857	1.68	0.227
Within sites	6.113	12	0.509		
Total	7.826	14

**Table:** Results of the local phylogenic community analyses using the site-specific termite pools. Pearson correlation (finer scale).

	Rainfall	Elevation	Temperature
NRI	0.354 (*p* = 0.195)	0.147 (*p* = 0.600)	−0.144 (*p* = 0.609)

**Table:** Estimates of the mixed effect model between NRI (using the site-specific termite pools) and the three abiotic variables rainfall, elevation, and temperature. The mixed model was insignificant (*p* > 0.087).

Variable	Estimate	Standard Error	df	t-Value	*p*-Value
Intercept	0.369	0.192	9	1.92	0.087
Rainfall	0.657	0.393	9	1.67	0.129
Temperature	0.490	0.593	9	0.82	0.430
Elevation	0.044	0.478	9	0.09	0.928

**Table A4:** Isotopic composition of δ^13^C‰ and δ^15^N‰ for each species of termites, soil, and litter.

Species	*n*	Delta Nitro		Delta Carbon	
		Mean	+/− SD	Mean	+/− SD
*Amitermes amicki*	5	5.65	0.57	−26.59	0.51
*Amitermes foreli*	10	7.57	2.68	−26.19	0.81
*Anoplotermes* sp1	11	11.31	2.11	−26.38	0.62
*Anoplotermes* sp2	10	10.35	1.12	−26.38	0.41
*Anoplotermes* sp3	10	10.81	2.54	−26.16	0.60
*Anoplotermes* sp4	11	10.76	2.53	−26.29	0.66
*Anoplotermes* sp5	6	8.00	1.15	−26.61	0.36
*Anoplotermes* sp6	10	7.51	0.96	−26.40	0.60
*Coptotermes testaceus*	8	2.23	1.26	−26.03	0.71
*Cornitermes* sp1	10	7.98	0.93	−26.65	0.57
*Cryptotermes colombianus*	2	1.35	1.76	−24.83	0.46
*Cryptotermes cylindroceps*	4	4.64	3.69	−25.56	1.02
*Heterotermes cardini*	11	3.52	1.42	−26.21	1.16
*Heterotermes convexinotatus*	6	2.47	0.60	−27.38	0.32
*Incisitermes schwarzi*	2	8.86	0.24	−28.23	0.25
*Microcerotermes arboreus*	10	1.86	0.96	−28.58	1.01
*Microcerotermes* sp1	11	1.42	1.09	−27.95	0.67
*Nasutitermes calliomorphus*	8	3.81	0.62	−28.51	0.82
*Nasutitermes corniger*	9	2.93	0.43	−27.69	0.62
*Nasutitermes dasyopsis*	7	3.30	1.35	−28.52	0.68
*Nasutitermes similis*	7	3.57	2.64	−27.95	0.75
*Nasutitermes* sp1	9	2.66	0.53	−28.40	0.51
*Nasutitermes* sp2	2	3.91	0.52	−29.45	0.26
*Nasutitermes* sp3	8	4.40	1.86	−28.00	0.93
*Neocapritermes longinotus*	10	8.64	1.36	−26.13	0.61
*Patawatermes* sp1	6	12.99	0.77	−26.04	0.60
*Proneotermes macondianus*	7	1.19	0.72	−27.17	0.53
*Rhinchotermes bulbinasus*	6	5.26	3.10	−27.13	0.43
*Ruptitermes* sp1	10	7.91	2.21	−26.57	0.69
*Tenuirostritermes* sp1	3	3.42	0.62	−28.99	0.27
*Termes* sp1	3	8.02	-	−24.57	-
*Termes hispaniole*	1	2.87	4.28	−27.23	3.06
*Litter*	75	4.0653	1.7535	−28.56	1.2804
*Soil*	15	5.98	1.4387	−27.34	0.9068

Table A5 Results of generalized linear models (GLM) that best fit the variation in isotope composition of δ^15^N ‰ in termites within tropical dry forest.

**Table:**

Source		df	F	*p*-Value
Intercept	Hypothesis	1	376.28	<0.001
	Error	27		
Sites	Hypothesis	2	4.78	0.030
	Error	12		
Feeding	Hypothesis	1	23.80	<0.001
	Error	212		
Subfamily	Hypothesis	3	12.22	<0.001
	Error	212		
Plots	Hypothesis	12	3.32	<0.001
	Error	212

**Table:** Tukey post hoc test between sites for δ^15^N‰.

(I) Sites	(J) Sites	Mean Difference (I–J)	Std. Error	*p*-Value
CE	CO	−0.99	0.34	0.013
	PT	−1.41	0.43	0.004
CO	PT	−0.42	0.42	0.580

**Table:** Tukey post hoc test between feeding groups for δ^15^N‰.

Feeding Groups		Mean Difference (I–J)	Std. Error	*p*-Value
I	II	−1.15	0.44	0.047
	III	−4.39	0.73	<0.001
	IV	−6.96	0.46	<0.001
II	III	−3.24	0.67	<0.001
	IV	−5.81	0.36	<0.001
III	IV	−2.57	0.69	0.001

**Table:** Tukey post hoc test between subfamilies for δ^15^N‰.

(I) Subfamily	(J) Subfamily	Mean Difference (I–J)	Std. Error	*p*-Value
Kalotermitinae	Rhinotermitinae	0.30	0.75	0.999
	Apicotermitinae	−6.77	0.65	<0.001
	Termitinae	−1.67	0.68	0.143
	Syntermitinae	−3.80	0.83	<0.001
	Nasutitermitinae	−0.28	0.68	0.998
Rhinotermitinae	Apicotermitinae	−7.07	0.53	<0.001
	Termitinae	−1.97	0.57	0.008
	Syntermitinae	−4.10	0.74	<0.001
	Nasutitermitinae	−0.58	0.56	0.903
Apicotermitinae	Termitinae	5.10	0.42	<0.001
	Syntermitinae	2.97	0.64	<0.001
	Nasutitermitinae	6.49	0.42	<0.001
Termitinae	Syntermitinae	−2.14	0.66	0.018
	Nasutitermitinae	1.38	0.46	0.031
Syntermitinae	Nasutitermitinae	3.52	0.66	<0.001

Table A6 Results of generalized linear models (GLM) that best fit the variation in isotope composition of δ^13^C ‰ in termites within tropical dry forest.

**Table:** GLM univariate for δ^13^C‰.

Source		df	F	*p*-Value
Intercept	Hypothesis	1	57,708.37	<0.001
	Error	37		
Sites	Hypothesis	2	5.32	0.022
	Error	12		
Feeding	Hypothesis	1	9.34	0.003
	Error	212		
Subfamily	Hypothesis	3	14.49	<0.001
	Error	212		
Plots	Hypothesis	12	2.13	0.016
	Error	212

**Table:** Tukey post hoc test between sites for δ^13^C‰.

(I) Sites	(J) Sites	Mean Difference (I–J)	Std. Error	*p*-Value
CE	CO	−0.12	0.12	0.560
	PT	−0.91	0.15	<0.001
CO	PT	−0.79	0.15	<0.001

**Table:** Tukey post hoc test between feeding groups for δ^13^C‰.

Feeding Groups		Mean Difference (I–J)	Std. Error	*p*-Value
I	II	1.28	0.15	<0.001
	III	−0.23	0.25	0.809
	IV	−0.13	0.16	0.863
II	III	−1.51	0.23	<0.001
	IV	−1.41	0.12	<0.001
III	IV	0.10	0.24	0.974

**Table:** Tukey post hoc test between subfamilies for δ^13^C‰.

(I) Subfamily	(J) Subfamily	Mean Difference (I–J)	Std. Error	*p*-Value
Kalotermitinae	Rhinotermitinae	−0.14	0.27	0.996
	Apicotermitinae	−0.21	0.23	0.944
	Termitinae	0.55	0.24	0.211
	Syntermitinae	0.26	0.29	0.950
	Nasutitermitinae	1.70	0.24	<0.001
Rhinotermitinae	Apicotermitinae	−0.07	0.19	0.999
	Termitinae	0.68	0.20	0.010
	Syntermitinae	0.40	0.26	0.656
	Nasutitermitinae	1.83	0.20	<0.001
Apicotermitinae	Termitinae	0.76	0.15	<0.001
	Syntermitinae	0.47	0.23	0.298
	Nasutitermitinae	1.91	0.15	<0.001
Termitinae	Syntermitinae	−0.29	0.23	0.827
	Nasutitermitinae	1.15	0.16	<0.001
Syntermitinae	Nasutitermitinae	1.44	0.23	<0.001
